# Complementarity of Clinician Judgment and Evidence Based Models in Medical Decision Making: Antecedents, Prospects, and Challenges

**DOI:** 10.1155/2016/1425693

**Published:** 2016-08-24

**Authors:** Zhou Lulin, Ethel Yiranbon, Henry Asante Antwi

**Affiliations:** ^1^Institute of Medical Insurance and Hospital Management, Jiangsu University, Zhenjiang, Jiangsu, China; ^2^School of Management, Jiangsu University, 301 Xuefu Road, Zhenjiang, Jiangsu, China; ^3^School of Graduate Studies, Ghana Technology University College, Private Mail Bag 100, Accra, Ghana

## Abstract

Early accounts of the development of modern medicine suggest that the clinical skills, scientific competence, and doctors' judgment were the main impetus for treatment decision, diagnosis, prognosis, therapy assessment, and medical progress. Yet, clinician judgment has its own critics and is sometimes harshly described as notoriously fallacious and an irrational and unfathomable black box with little transparency. With the rise of contemporary medical research, the reputation of clinician judgment has undergone significant reformation in the last century as its fallacious aspects are increasingly emphasized relative to the evidence based options. Within the last decade, however, medical forecasting literature has seen tremendous change and new understanding is emerging on best ways of sharing medical information to complement the evidence based medicine practices. This review revisits and highlights the core debate on clinical judgments and its interrelations with evidence based medicine. It outlines the key empirical results of clinician judgments relative to evidence based models and identifies its key strengths and prospects, the key limitations and conditions for the effective use of clinician judgment, and the extent to which it can be optimized and professionalized for medical use.

## 1. Introduction

One vocation that requires the personal knowledge, skills, and judgment of service providers is the medical profession. These elements are required by clinicians to protect and restore the wellbeing of people with the greatest possible firmness [1, 2]. At the heart of the doctors connoisseurship is how their individual expertise and skills are deployed for effective clinical judgment and this is as important as the doctor's technical capability in carrying out the core medical procedures itself [3]. According to [4] doctors develop skills to make effective medical judgment through experience from practice and knowledge shared with comrades, critical analysis, continuous research, and ongoing professional development. This extends to all medical areas including diagnosis, therapy, prognosis, communication, and other medical decision making. However, clinician judgment has its own critics and is sometimes harshly described as notoriously fallacious and an irrational and unfathomable black box with little transparency [5, 6]. The past decade has seen the emergence of several new investigations and theories about applying clinical judgment but most of them have been restricted to its role in communication, diagnosis, prognosis, and other medical decision making without much discussion on their validity, potential competence, reliability, susceptibility to error and bias, and the extent to which it can be optimized and professionalized for general use [7, 8]. This review revisits and highlights the core debate on clinical judgments and its interrelations with evidence based medicine. It outlines the key empirical results of clinician judgments relative to evidence based models and identifies its key strengths and prospects and the key limitations and conditions for the effective use of clinician judgment.

## 2. Emergence of Evidence Based Medicine

Early accounts of the development of modern medicine suggest that the clinical skills, scientific competence, and doctors' judgment were the main impetus for treatment decision, diagnosis, prognosis, therapy assessment, and medical progress [9]. However, with the rise of contemporary medical research, the reputation of clinician judgment has undergone significant reformation in the last century as its fallacious aspects are increasingly emphasized relative to the evidence based options. Critics of clinical judgment presumes that it cannot go beyond a simple* post hoc ergopropter hoc* but can at best achieve simple, intuitive, low-quality correlational statistics [10, 11]. Coupled with an increasing numbers of judgmental errors on the part of doctors, a primary mission was initiated “to guard against any use of judgment” [12, 13] while emphasis moved to the exploration and use of clinical trials.

Since the 1960s the “antiguessing” theory of evidence based medicine (EMB) currently practiced globally by clinicians has dominated medical practice and associated decision making following series of publications by Alvan Feinstein, Archie Cochrane, John Wennberg, David Eddy, David Sackett, and so forth, [14–16]. As an optimized clinical decision making approach, EBM emphasizes evidence from well designed and executed research as the fulcrum of all clinical decisions. Even though all medicine based sciences have some degree of empirical validation, EMB goes further by classifying evidence by its epistemological strength and recommends only the strongest types (coming from meta-analyses, systematic reviews, and randomised controlled trials) [17]. To consolidate evidence based medicine, disease severity scoring systems such as APACHE II and mathematical methods like likelihood test, seasonal autoregressive integrated moving average [18–20], other time series regressions [21–23], Cox hazard models [19, 20, 24], exponential smoothing [25–27], and so forth have been applied to patient data to ensure accurate forecast of future patient conditions and other decision scenarios.

Despite the successful application of these traditional statistical models in healthcare, the complexity of the human body, the multidimensional and nonlinear nature of biological systems, and clinical characteristics limits their predictive ability. With the emergence of data mining, Artificial Neural Networks has been experimented to support evidence based medicine in assessing [18, 20, 28] and predicting [29–31] more complex biological systems and medical scenarios with greater degree of accuracy over the conventional statistical models albeit their weaknesses. While the important place and role of EBM in contemporary medical practice are strongly represented in modern healthcare literature it also has its fair share of criticisms. For example the authors in [32] criticise EMB for its restricted process of evidence collection and approval. They contend that “EMB sometimes suffer from a ‘Central Control' phenomenon as a few chosen experts are tasked with the responsibility of digging out evidence, then instruct others on how to interpret and utilise the evidence.”

Moreover [33] argues that the quantitative results produced by EBM research especially from randomised controlled trials may be irrelevant for some treatment situations while racial minorities and people with comorbid diseases which are usually underresearched may limit the generalisability of randomly controlled trials. Reference [22] reports disparities between treatments effectiveness reported from randomised controlled trials and those achieved in routine clinical practice and population based research which EBM champions may not apply on a patient by patient basis. Thus, in most instances, the knowledge acquired from clinical research studies to design evidence based standards fails to directly address clinical questions regarding what is best for the patient at hand.

Within the last decade, medical forecasting literature has seen significant attempt to revisit the role of clinician (doctors) judgment in medical decision making as a complement of EBM due to its practical limitations. Reference [34] stresses that the grand attempt to discredit the use of personal judgment by clinicians in the 1960s was not based on systematic investigations but on selectively procured sample of judgmental error or sometimes anecdotal examples of error and naivety on the general low esteem of personal cognition in the times of neopositivist [16, 30, 35] and fallibilist [30, 36, 37] epistemologies. Reference [38] and other “radical” advocates of clinical judgments emphasize that the experience of different expert (clinicians) can complement EBM in specific medical decision scenarios such as when treating new illness with limited statistical data, in prognosis of survivability of a particular disease [39], and when there are few records of patient data with given symptoms. In that case making available the judgment or experience of physicians who have encountered several such cases during years of practice can provide valuable additional information for decision making.

Reference [23] affirms this by proposing that in some sense experts are human measuring instruments. Just as a sensor can measure a patient's blood pressure, temperature, and so forth, the experience of a medical expert can supplement these measurements in diagnosis and prognosis. This argument is reasonable to the extent that experienced and competent professionals rely on both explicit factual evidence and their tacit knowledge before making any decision [40–44]. Any competent practitioner worth his or her profession is disposed to make several judgments of which the specific or adequate criteria cannot be easily expressed and equally displays skills whose rules and procedures cannot be explicitly stated. In this case he or she depends on tacit recognitions, judgments, and skillful performances to draw conclusions which are mostly accurate [19, 22, 45, 46]. Thus “there is a clamour to represent individual variety in medical prognosis and corresponding decision making through alternative but accurate prediction approaches” and should be provided a platform for presentation.

However a more conservative view in the clamour to represent clinical judgment in the medical decision making process has emerged to help control potential clinician abuse. Reference [24] rather advocates for what they call a “cybernetic variety” that deemphasizes individual doctor's judgment and rather proposes the creation of a “pool of experience” from which clinicians can draw experiential information when faced with context specific medical dilemma. In this way using “crowd wisdom” approach instead of “individual wisdom” is presented as a more credible option to complement EBM and help gather all available knowledge, experience, possible alternatives, or bits of information from experts together to treat specific healthcare cases [47, 48].

Reference [29] espouses the innate wisdom of the crowd as opposed to individuals in the story of “cleaning the crystal ball.” This story discusses the challenges of prediction using the old game of estimating the number of jelly beans in a jar. In a 1987 study conducted by Professor Jack Treynor, 56 students were asked to provide estimates of how many jelly beans were in a jar. The mean guess of the students was 871, representing a 97.6% level of accuracy, with only one of the 56 estimates getting closer to the actual value of 850; see [49, 50]. In support of Treynor's work, a similar study conducted by the researcher, again sampling estimations from 56 students showed a similar level of accuracy of 98.7%.

According to [51], using “crowd wisdom” in medical decision making is driven and embodied by Ashby's Law which is applicable in many forms. “Ashby's Law” stipulates that the minimum amount of information needed to give an accurate answer is exactly the amount needed to specify the problem. This is interpreted as if the question has lot of variety the answer too will have the same amount of variety. A complicated question will obviously not have a simple answer either. In clinical decision, management of a complex fracture in patient with multiple comorbidities in a resourcefully challenged situation cannot be resolved by “Cookbook” approach presented by evidence based medicine. Thus if we need an answer to a complex situation, more information will be needed on a large scale and pooling the “wisdom of the medical crowd” will be more effective than a controlled approach [52].

## 3. Origins of Wisdom of the Crowd Theories

Wisdom of the crowd is the basis of modern prediction markets which utilise the knowledge of a pool of individuals to help forecast questions of importance to organisations in a timely manner. In 1906, scientist Francis Galton's curiosity for individuals' physical and mental qualities, in addition to his obsession for animal breeding, led him to become a seminal author of work on the “wisdom of crowds” [53]. During what was originally intended as a leisurely day out for Galton at the annual West of England Fat Stock and Poultry Exhibition in Plymouth, he stumbled across a weight-judging competition where members of the public, skilled and unskilled alike in the task of judging the weight of a Fat Ox, were paying sixpence to guess the Ox's weight in the knowledge that the closest individual estimate to the actual weight of the Ox, once it had been “Slaughtered and Dressed,” would win a prize (see [54]). Surowiecki [55] narrates the story of Galton's decision to turn the competition into an “impromptu” experiment. Galton's initial aim was to in fact affirm his belief that “the stupidity and wrong-headedness of many men and women was so great as to be scarcely credible” [55]. Yet Galton was to be surprised by his findings. He collated all of the 787 legible estimates and calculated the mean of these estimates, acquiring a figure of 1,197 pounds, one away from the correct weight of 1,198 pounds, an error of only 0.09% [56].

Around the time Galton published his findings, the traditional literature relating to collective judgments as opposed to those of the individual was somewhat to the contrary [50, 57]. Charles Mackay had published on the “Madness of Crowds” in his 1841 magnum opus in which he stated that “men, it has been well said, think in herds; it will be seen that they go mad in herds, while they only recover their senses slowly, one by one.” Similarly the speculator Bernard Baruch in the forward of the republication of Mackay's work wrote “anyone as taken as an individual is tolerably sensible and reasonable-as a member of a crowd, he at once becomes a blockhead” (1932). Supporting the views of Charles Mackay and Bernard Baruch were authors such as Thoreau [58], Nietzsche [59], and Carlyle (see [60–62]) to name a few. Perhaps the harshest critic of the wisdom of crowds was the French psychologist Gustave Le Bon in his 1895 publication study [63, 64]. Le Bon was an advocate for the belief that individual opinions are superior to those of the crowd and was also a ruthless critic of his antecedents such as Herbert Spencer [65, 66]. Le Bon as cited in [67] utilised a chemical analogy to portray his standing that individuals collaborating in a crowd are like “certain elements, combined to form a new body possessing properties quite different from those of the bodies that have served to form it.” LeBon described any assembly of people (no matter their true intention) “an organised crowd.” He stated that “how much” an isolated individual “differs” from a crowd of which they are a part can be “easily measured”, yet he does not provide examples of measurements other than to declare that juries return verdict to which each of the individual jurors would disapprove while also deeming that “parliamentary assemblies adopt laws and measures of which each of their members would disapprove in his own person” [68–70].

Despite the initial setback, the use of crowd wisdom to build prediction markets gained momentum again in a series of articles written by Robin Hanson (see [71, 72]) yet prior to this in 1988 the earliest known application of crowd wisdom for prediction was initiated by the Iowa Electronic Markets (IEM). These markets were aimed at studying market dynamics while acting as a predictive mechanism for the outcome of elections [56]. Since their introduction in 1988, the IEM have proved to be “highly consistent” returning “remarkable accuracy” outperforming traditional and often more publicly appraised political polls over three-quarters of the time [73].

Within corporate firms, crowd wisdom has been used to construct prediction markets to produce outcomes to numerous issues: numerical forecasting, decision making, and risk management to name a few. Whether used to predict demand for a good or service, to assist management to decide which product to produce, or to develop ideas as to the level of exposure within a marketplace, crowd wisdom has been an extremely effective tool for decision makers, when used in a functional environment correctly [74]. In 1996 HP conducted its first field application of crowd wisdom for prediction requesting that 26 “involved executives” forecast the future demand for a family of products [75]. Despite the crowd not being as large or having perhaps diversity as Surowiecki may have wished, the prediction error was far lower than the official forecast error for six of the eight comparable events [75]. In the example above, HP's incentive to use the aggregating power of the crowd of executives was to test the accuracy of their usual forecasts which was often developed by one “expert” manager relative to forecast generated by the whole set of managers. In a similar market to that of HP, academics [56] attempted to find a mechanism in which “a relatively small group of novice participants could achieve the same results as experts that generate pricing decisions (within the airline industry) by engaging in a costly and intelligent process of analyzing quantitative and qualitative data.” Conducting their study based on the airline El Al, the academics found that through the use of a simple constituted prediction market or crowd, consisting of only 51 participants, they could produce a pricing structure that was only 0.4% or $3.50 different from the pricing set by the airline [56].

Over the past decade, General Electric (GE), one of the world's most powerful organisations, held their own internal “Ideas Bank” [76] where a Virtual Concept Testing mechanism was set up in which the opinions of a crowd are aggregated to determine the products or ideas they most highly favour as well as the predicted trading price of each of the product [77]. GE used this crowd information aggregation in 2006 to elicit and rank-order technology and product ideas from across the subbusinesses. They, like a number of leading academics, feel that such markets offer more promise than more traditional methods such as surveys, suggestion boxes, and brainstorming sessions [78].

## 4. Use of Crowd Wisdom in Medical Literature

Instances of application of crowd wisdom theory in the medical literature are presented under different healthcare decision scenarios with conflicting outcomes. In 1976, [79] randomly selected a sample of 65 general practitioners and 78 medical and surgical gastroenterologists to predict the likely current state of a cohort of 227 patients first diagnosed with duodenal ulcer in 1963 in hospitals and general practice. This was after the experts had extensively reviewed the medical profile of each patient. At the time the actual state of the 227 patients showed that 50 patients had died, 57 had been medically treated with no symptoms, 44 had mild symptoms, and 34 had been treated surgically while 19 of them had more severe symptoms. The remaining 12 had emigrated. The study noted that cases that had been diagnosed in hospitals had a more severe prognosis than those diagnosed in general practice. The individual prediction deviation of the experts was very wide showing that individual prediction estimate was less reliable. However, the mean prediction level by all doctors differed marginally from the actual estimates suggesting the reliability of collective experience of the medical profession. The study also found out that the general practitioners, surgeons, and physicians showed insignificant systematic differences, a reflection of the differences in the types of patients they treat.

Reference [80] has also evaluated the use of crowd in prognostic scenarios when they studied the accuracy of crowd wisdom technique in predicting long-term prognosis of patients with coronary artery disease. This study compared whether the prognosis of five senior clinical cardiologists (familiar with case summaries of 100 randomly sampled patients with significant coronary disease selected from a large series of medically treated patients) was better than the predictions of data-based multivariable statistical model (Cox regression models). Differences in the collective prognosis as well as the individual doctor prognosis were measured. Each of the five cardiologists predicted a one- to three-year survival and infarct-free survival probability of the 100 patients and 50 patients appeared in multiple samples of interphysician variability. A comparison of the corresponding outcome probability with the computed Cox Hazard Proportion Regression showed that the latter's prediction accuracy was better than the correlation between doctor prediction and actual patient outcome. The statistical model predicted a three-year survival with a rank correlation of 0.61 while that of the collective view of the doctors was 0.49 (doctors). The statistical models' three-year infarct-free correlation prediction outcome was 0.48 while that of the doctors was only 0.29. This study showed that carefully developed statistical models from collected data can provide better prognostic prediction than the experience of clinician made from case summaries.

Reference [81] has applied crowd wisdom technique to predict survivability of patients in the daily flow of ICU patients. In that study two clinicians and some nursing sisters working in the intensive care unit (ICU) were asked to indicate the number of the patients in the department who will survive the current condition. Each patient was assessed and classified into one of two groups, namely, “unknown outcome” or “will die.” The daily predictions were then compared with computerised trend analysis of daily acute physiology and chronic health evaluation (APACHE II) scores. These scores were corrected to account for confounding factors such as the presence and duration of major organ system failure. The comparative analysis of the prediction outcome and that of the actual hospital outcome showed that doctors and nurses predicted the death rate falsely at 16.6% individually but collectively their false prediction was reduced to 7.7%. The death rate predicted by the computer generated models was rather minimal. Moreover the patients that were predicted to die by nurses and doctors were not identical to those predicted to die by the computer model. Finally a confirmatory test showed that the sensitivity of prognosis of doctors and nurses was 20% and this is lower than the computer models.

The aggregated wisdom of a small group of virologist and microbiologists was solicited by [82] to predict the possible influenza activity between 2 and 4 weeks. Their aggregate prediction was more accurate than the predictions derived from historical data for the same activity and the individual expert predictions. This study revealed that beyond predicting seasonal influenza, collective clinician experience is useful in microbiology for planning and managing outbreak of infectious diseases.

Another healthcare application of the innate wisdom in crowd knowledge is by [17] who aggregated crowd knowledge from the social media to strengthen the surveillance capacity of influenza in Germany. The study aggregated crowd's behaviour and comments on Twitter during the world's largest Enterohemorrhagic* Escherichia coli* (EHEC) outbreak in Germany in May 2011. These recorded aggregated crowd's behaviour helped to document the critical messages of users which triggered signal detection alarms ahead of highly established early detection systems such as by MedISys [17].

Outside the clinical environment, the crowd wisdom techniques have been used in other healthcare forecasting scenarios such as healthcare service demand. In their ground breaking work, [83] highlighted findings of a study conducted at the Royal Devon and Exeter Hospital where the wisdom of the crowd technique was employed to forecast service demand. Based on the outcome of aggregated information collected from sixty-five participants over a period of one week, the effectiveness of prediction markets was confirmed as a strong forecasting tool. In this premier study participants were asked to estimate the daily number of patients arriving at the Royal Devon and Exeter Hospital. The tool was more effective in forecasting hospital service demand with an error of 0.3% but less effective in interdepartmental predictions [83].

The work of [11] also presents another interesting dimension of how crowd wisdom techniques compare to individual clinician wisdom and computerised and statistical predictive models in medical forecasting scenarios. After the discharge of selected patients from a medical facility, physician house officers were asked to predict the likelihood of these patients returning for follow-up visits and the amount of prescribed drugs they were likely to take. This was then benchmarked against a patient compliance test conducted on 187 patients discharged from the same medical facility. Reference [11] reports that only 35% of patients predicted by physicians to revisit actually did return and half of their noncompliance predictions were incorrect. Regarding prediction of medication compliance, less than half of individual predictions correctly discriminated between noncompliant and compliant patients while three-fourths of their collective predictions of noncompliance were accurate. On the basis of this argument the researchers argued that physicians have clinical diagnostic limitations if left unaided hence the need to support physical decision making through continuous professional education and skills in diagnosing and managing sociobehavioural aspects of their profession.

## 5. Prospects of Medical Use of Wisdom of the Crowd

Reviewing the reported application of clinician judgment in medical decision making as discussed in the empirical studies above brings out some useful conclusions about the extent of their applicability. It is obvious that the collective judgment of the various clinicians in these studies proves more reliable than the individual doctor judgment. This may indicate some value in crowd wisdom over individual doctor judgment but not sufficient information to make concrete generalisations. Secondly, the available empirical works that evaluate clinician judgment and statistical models present conflicting outcome of superiority and inferiority under different decision context.

More interestingly, a dominant trend shows that while crowed techniques are useful, they appear to be more effective when used under desirable conditions and in conjunction with the right statistical evaluation (a case that supports its complementary role with evidence based models). Generally, it seems that medical decision making (especially forecasting, diagnosis, therapy, prognosis, communication, etc.) can benefit from crowd wisdom for the temporal accumulation of medical information over time which may lead to the development of a “Swarm Intelligence” algorithm where pieces of information are brought together to form a part of the “Swarm” to stimulate intelligent informed behaviours in medical decision making [11, 12].

Reference [19] explains swarm intelligence as a discipline that deals with collective behaviours of individuals that are coordinated by decentralised and self-organising control systems. A “medical swarm” (as a collective database of experience and knowledge of expert clinicians) has the potential to benefit from an important property of swarm intelligence system. It can act in a coordinated manner despite the lack of leadership or an external controller. Many examples can be seen in the nature of swarms that perform some collective behaviour such as the ant colony, without any individual who controls the group or is to be aware of the overall behaviour of the group [31]. In these swarms, each individual has a stochastic behaviour that depends on its local perception of the community hence possible to design a system of swarm intelligence that is scalable (maintain its function, while increasing its size without the need to redefine how its parts interact), parallel, and fault tolerant.

Thus similar to the clustering behaviour of ants, nest building behaviour of wasps and termites, crowding and schooling in birds and fish, ant colony optimization and particle swarm optimization, the wisdom of the medical crowd can be harnessed for diagnosis, prognosis, other medical decision scenarios, and so forth. The above concepts of swarm intelligence are already inspiring new initiatives in medical literature and practice such as the online medical forum by the Indian Orthopaedic Research Group (IORG) and similar ones in other parts of the world [84]. In these forum surgeons presents the clinical and radiological details of their cases to elicit comments from other clinicians based on their personal experience and familiarisation of the current literature on the subject.

This helps clinicians to obtain different perspectives on a variety of topical issues affecting their practice by quickly sharing knowledge and effectively using “wisdom of the medical crowds” [85]. The Journal of Orthopaedic Complications and the Orthopaedic Case Bank have also been launched by the Indian Orthopaedic Research Group (IORG) to accept only complications or complicated cases to elicit discussions by the community of orthopaedic surgeons. With time this “Bank” can grow and become a warehouse with a variety of cases that can be grouped together and searched simultaneously by individual clinicians and others who need them [86]. The next step is to regularize the forums and develop a good publishing format and start publishing these rich case discussions, either as a part of a journal or in other citable online formats in public domains. This will make this information available to more viewers and also to generations to come as a template of current thought process. Algorithms can be developed based on case characteristics to find the nearest neighbour and also to provide recommendation based on data in the “Bank.”

## 6. Challenges of Medical Use of Wisdom of the Crowd

Despite the potential advantages of clinician judgment and crowd wisdom in medical forecasting, its usefulness is attenuated by several challenges that must be managed with the greatest possible firmness. A more engaging discussion of the limitations of clinician judgment hence the occasional challenges in relying on wisdom of the crowd theories in medical decision making is provided by [10]. The author explains that, in using wisdom of the crowd techniques in medical forecasting, it must be noted that doctors can make suboptimal diagnostic and treatment decisions. With reference to [23] comparison of the doctor's opinion as human measuring instrument that can supplement medical sensors and devices, it must be noted that these devices are usually imprecise (have some margin of error in their results). This is the same as the judgment of the clinician which is also imprecise when it is used for diagnosis, prognosis, therapy, and so forth. Thus there is a limitation in depending on doctor's judgment or experience for medical decision making since human judgments are subject to biases.

According to [87] a number of biases can affect the ways in which doctors gather and use evidence in making diagnoses in particular. Biases also exist in how doctors make treatment decisions once a definitive diagnosis has been made. These biases are not peculiar to the medical domain but, rather, are manifestations of suboptimal reasoning to which people are susceptible in general. Nonetheless, they can have potentially grave consequences in medical settings, such as erroneous diagnosis or patient mismanagement [86]. As benchmarks, any medical “wisdom” generated from the “medical crowd” must give careful consideration to the vulnerability of doctors' reasoning to a number of biases that can lead to errors in diagnosis and treatment. That is judgment errors must be eliminated from doctor's opinions in order to become more reliable or accurate tools for medical prediction [88].

Even though there are no surefire methods to eliminate or alleviate the biases that affect individual doctor biases in medical decision making there is the need to incorporate formal decision analytic tools to improve the quality of doctors' reasoning and enhance their reliability as prognostic tools to complement current evidence based models. Reference [89] suggests that incorporating fuzzy algorithms and Bayesian probabilistic models can help to alleviate prediction errors or biases from doctor's judgment.

Secondly the effectiveness of crowd wisdom in medical decision making especially in medical forecasting is also largely dependent on the prevalence of certain conditions including diversity, independence, decentralisation, and motivation of the contributors. Ever since the work of Galton, mathematical models have been used to examine the accuracy of simulations of crowd wisdom with psychologists, econometricians, and financiers alike attempting to ascertain the conditions under which crowd wisdom is capable of achieving reliable outcomes [90].

From work undertaken by Hogarth [91] and Makridakis and Winkler [92] as cited by [93] it is inferred that if a crowd's judgment contains “signal-plus-noise,” averaging judgments will cancel out noise thus revealing a signal [94, 95]. As Surowiecki [55] states, the real key to “tapping” crowd wisdom is not so much perfecting the method used but is satisfying the conditions that groups require in order to be “smart.” The first condition as laid out by Surowiecki relates to the diversity of the crowd (also see [90, 96, 97]). Diversity relates not to culture or ethnicity but to knowledge and approach. Political scientist Scott Page's [98] as cited in [97] belief was that intelligence alone could not provide nor guarantee different perspectives on a problem and thus supported March's [99] claim that the effect of making a group smarter “does not come from the superior knowledge of the average new recruit. Recruits are, on average, less knowledgeable than the individuals they replace. The gains come from their diversity.”

Although this concept may perhaps be hard to grasp initially, it can be supported by earlier work from [100] and also cited in [32] which discussed the proficiency of an expert chess player in comparison to an amateur. They demonstrated that, showing the two players of differing ability a game in progress, the expert will be able to map out the game from memory yet the amateur could not; thus the best decision may be to “chase the expert” [32] and not distort the expert view with that of an amateur. This however is a situation that changes when the board is in a haphazard state, as then the expert is unable to recreate the spread of the pieces making his expertise no more valuable compared to that of the amateur. This led Chase and Simon [100] to conclude that the use of expert knowledge is indeed “spectacularly narrow.”

The second condition for effective dependence on crowd wisdom in medical decision making is independence of thought. Independence of thought and estimation when gathering crowd wisdom have long since been an intensely discussed subject within the literature (see [55, 101]). Although evidence abounds in the current literature to show that individual judgment are usually accurate, [55] argues that individual judgment can become more accurate if other people influences the individual. Independence of estimates, such as those collected by Galton, is where individuals configure their own estimations or views based solely on what economists term as private information.

Independence is important because any error that one person may make in their estimate will not be passed on to any other person's estimate, thus avoiding systematic bias. References [90, 102] argue, however, that independence does not eradicate the possibility of systematic error due to the fact that many systematic biases arise only among populations (e.g., college students) in which participants lack the requisite knowledge. In addition it has also been reported that systematic errors may occur in environments where full independence and diversity exist as a result of overestimation or optimism biases [103, 104].

The approach taken by authors such as Asch [105], Festinger [106], Galton [107], and Treynor [108] as reported by [109] support of independent estimations formulating a more accurate mean group prediction is one which is contrasted by works, albeit fewer in number, such as [97, 110], who argue in favour of interaction between estimators. Both approaches have shown their value within the mentioned studies and likewise both methods have been challenged as to their effectiveness. It is however a given that the psychological approach to making rational choices is questioned throughout both the economic and psychological academia (see [111]) and yet despite his statement of the importance of independence [55] does however write, “independence is hard to come by. We are autonomous beings, but we are also social beings…we want to learn from each other, and learning is a social process.”

Thirdly there is the need to ensure decentralisation when drawing on the collective wisdom of medical experts of decision making. Relating back to the work of Hayek, tacit knowledge, knowledge that cannot easily be relayed or abridged for the benefit of others due to its specific nature, can be seen as a crucial principle as to why decentralisation is seen as a condition for successful information aggregation among crowds [55]. Decentralisation promotes the views of Adam Smith on specialisation, allowing those who have specific knowledge to express it independently yet amongst coordinated activity bound by a question or unknown outcome. The reason for the importance of decentralisation is that should information holding agents be too distant from one another in physical or colloquial form, problems can occur as information sourced by one member of a decentralised system cannot be waylaid through to the rest of the system, causing potential valuable information to be lost [55]. This likelihood of not all information being aggregated from decentralised participants has been named by [112] as the “inevitability of decentralised decision making.”

The provision of an incentive mechanism has been described as critical “since people and as such doctors may invest more thought and energy into expressing their opinion when they have a strong incentive to do so” [113]. Although, in practice in some cases, incentives to participants have been offered, whether these are monetary or of other nature, in the cases of Treynor's bean jar and the Hollywood Stock Exchange, accurate results have been recorded despite the only incentive for participants being pride in the accuracy of their proposal. Incentives can be provided by a number of differing means. Galton's Ox experiment for example requested sixpence to enter a guess and be in with a chance of winning a prize (also see [94]). This is likely to have only encouraged those who felt they were capable of estimating relatively accurately to enter, which creates a kind of market entry barrier. In advancement on Treynor's bean jar experiment the researcher offered a prize for the most accurate estimate from the 56 students who entered guesses. This apparently improved accuracy of the study by 1.1%; however, it must be noted that this may not be the overriding reason for the discrepancy.

## 7. Conclusions

The study has analyzed the crowd wisdom as a medical decision making tool and other healthcare related scenarios presented in the extant literature. Based on the discussion, this paper supports the view that crowd wisdom models can be utilised as a successful decision making tools (prognosis, diagnosis, therapy recommendation, health service planning, and so forth). When utilised correctly it can also be a tool of enormous power for several areas of public health decision making including patient flow, bed allocation, transport scheduling, staff scheduling, supply chain management, and menu services. It can provide more accurate forecasts than traditional methods assisting staff planning as well as reducing costs to the hospitals. Naturally, problems will develop with these forms of practical markets as with any evolving technique, but, from the evidence this paper has discussed, at least crowd wisdom techniques will become much more commonplace in the future for healthcare organisations to reduce costs and free up valuable resources, ultimately bettering quality of health service. There is the need for more empirical studies on the subject with larger sample size and in different healthcare decision scenarios. This would assist with weight averaging and also, depending upon the nature of the study, endorse or disprove numerous issues raised within this and other studies, such as crowd or expert anomalies. In order to examine the dispute of the effects of time-scales in predictions of experts, further studies covering wider spans of time could be conducted. In an ideal further study within a hospital environment, one should be able to wager on estimates as a way to weight predictions. This would also provide more incentive. More variables could also be investigated in order to increase the probability *F*-Statistics of regression models derived from crowd wisdom techniques in healthcare environment. Finally, a cascade in the form of a Delphi method could be utilised to create a more sophisticated swarm of intelligence or prediction market.

## References

[1] Gao C., Song J.-S., Xiong J., Xue X.-H., Shang T.-G. (2012). Standard research on the quality of published medical case literature from Treatise on Febrile Diseases based on the Delphi method. *China Journal of Traditional Chinese Medicine and Pharmacy*.

[2] Spiby J. (1988). Advances in medical technology over the next 20 years. *Journal of Public Health*.

[3] Nimgaonkar A., Karnad D. R., Sudarshan S., Ohno-Machado L., Kohane I. (2004). Prediction of mortality in an Indian intensive care unit: comparison between APACHE II and artificial neural networks. *Intensive Care Medicine*.

[4] Yu T., Chen S.-H. (2012). Agent-based modeling of the prediction markets for political elections. *Multi-Agent-Based Simulation XII: International Workshop, MABS 2011, Taipei, Taiwan, May 2–6, 2011, Revised Selected Papers*.

[5] Ohno-Machado L. (2001). Modeling medical prognosis: survival analysis techniques. *Journal of Biomedical Informatics*.

[6] Akins R. B., Tolson H., Cole B. R. (2005). Stability of response characteristics of a Delphi panel: application of bootstrap data expansion. *BMC Medical Research Methodology*.

[7] Bederman S. S., McIsaac W. J., Coyte P. C., Kreder H. J., Mahomed N. N., Wright J. G. (2010). Referral practices for spinal surgery are poorly predicted by clinical guidelines and opinions of primary care physicians. *Medical Care*.

[8] Ohi G., Kai I., Kobayashi Y. (1987). AIDS prevention in Japan and its cost benefit aspects. *Health Policy*.

[9] Uchino S., Bellomo R., Goldsmith D., Bates S., Ronco C. (2006). An assessment of the RIFLE criteria for acute renal failure in hospitalized patients. *Critical Care Medicine*.

[10] Bornstein B. H., Emler A. C. (2001). Rationality in medical decision making: a review of the literature on doctors' decision-making biases. *Journal of Evaluation in Clinical Practice*.

[11] Mushlin A. I., Appel F. A. (1977). Diagnosing potential noncompliance: physicians' ability in a behavioral dimension of medical care. *Archives of Internal Medicine*.

[12] Christensen C., Elstein A. S. (1991). Informal reasoning in the medical profession. *Informal Reasoning and Education*.

[13] Kittur A., Chi E., Pendleton B. A., Suh B., Mytkowicz T. (2007). Power of the few vs. wisdom of the crowd: wikipedia and the rise of the bourgeoisie. *World Wide Web*.

[14] Jones R. P. (2010). Myths of ideal hospital size. *Medical Journal of Australia*.

[15] Bradbury A. W., Adam D. J., Bell J. (2010). Bypass versus Angioplasty in Severe Ischaemia of the Leg (BASIL) trial: a survival prediction model to facilitate clinical decision making. *Journal of Vascular Surgery*.

[16] Smart K. M., Blake C., Staines A., Doody C. (2010). Clinical indicators of ‘nociceptive’, ‘peripheral neuropathic’ and ‘central’ mechanisms of musculoskeletal pain. A Delphi survey of expert clinicians. *Manual Therapy*.

[17] Diaz-Aviles E., Stewart A., Velasco E., Denecke K., Nejdl W. Epidemic intelligence for the crowd, by the crowd.

[18] Jones S. S., Thomas A., Evans R. S., Welch S. J., Haug P. J., Snow G. L. (2008). Forecasting daily patient volumes in the emergency department. *Academic Emergency Medicine*.

[19] Bassi P., Sacco E., De Marco V., Aragona M., Volpe A. (2007). Prognostic accuracy of an artificial neural network in patients undergoing radical cystectomy for bladder cancer: a comparison with logistic regression analysis. *BJU International*.

[20] Biglarian A., Hajizadeh E., Kazemnejad A., Zali M. R. (2011). Application of artificial neural network in predicting the survival rate of gastric cancer patients. *Iranian Journal of Public Health*.

[21] Amiri Z., Mohammad K., Mahmoudi M., Parsaeian M., Zeraati H. (2012). Assessing the effect of quantitative and qualitative predictors on gastric cancer individuals survival using hierarchical artificial neural network models. *Iranian Red Crescent Medical Journal*.

[22] Zhu L., Luo W., Su M. (2013). Comparison between artificial neural network and Cox regression model in predicting the survival rate of gastric cancer patients. *Biomedical Reports*.

[23] Ansari D., Nilsson J., Andersson R., Regnér S., Tingstedt B., Andersson B. (2013). Artificial neural networks predict survival from pancreatic cancer after radical surgery. *The American Journal of Surgery*.

[24] Wang J., Li M., Hu Y.-T., Zhu Y. (2009). Comparison of hospital charge prediction models for gastric cancer patients: neural network vs. decision tree models. *BMC Health Services Research*.

[25] Xu Y., Selaru F. M., Yin J. (2002). Artificial neural networks and gene filtering distinguish between global gene expression profiles of Barrett's esophagus and esophageal cancer. *Cancer Research*.

[26] Wang C.-H., Mo L.-R., Lin R.-C., Kuo J.-J., Chang K.-K., Wu J.-J. (2008). Artificial neural network model is superior to logistic regression model in predicting treatment outcomes of interferon-based combination therapy in patients with chronic hepatitis C. *Intervirology*.

[27] Uys A., Rapoport B. L., Fickl H., Meyer P. W. A., Anderson R. (2007). Prediction of outcome in cancer patients with febrile neutropenia: comparison of the multinational association of supportive care in cancer risk-index score with procalcitonin, C-reactive protein, serum amyloid A, and interleukins-1*β*, -6, -8 and -10. *European Journal of Cancer Care*.

[28] Spelt L., Nilsson J., Andersson R., Andersson B. (2013). Artificial neural networks-A method for prediction of survival following liver resection for colorectal cancer metastases. *European Journal of Surgical Oncology*.

[29] Burke H. B., Goodman P. H., Rosen D. B. (1997). Artificial neural networks improve the accuracy of cancer survival prediction. *Cancer*.

[30] Chen L.-J., Lian G.-P., Han L.-J. (2007). Prediction of human skin permeability using artificial neural network (ANN) modeling. *Acta Pharmacologica Sinica*.

[31] Shi H.-Y., Tsai J.-T., Chen Y.-M., Culbertson R., Chang H.-T., Hou M.-F. (2012). Predicting two-year quality of life after breast cancer surgery using artificial neural network and linear regression models. *Breast Cancer Research and Treatment*.

[32] Soll J. B., Larrick R. P. (2009). Strategies for revising judgment: how (and how well) people use others' opinions. *Journal of Experimental Psychology: Learning Memory and Cognition*.

[33] Webster J. G., Ksiazek T. B. (2012). The dynamics of audience fragmentation: public attention in an age of digital media. *Journal of Communication*.

[34] Shi H.-Y., Lee K.-T., Lee H.-H. (2012). Comparison of artificial neural network and logistic regression models for predicting in-hospital mortality after primary liver cancer surgery. *PLoS ONE*.

[35] Graefe A., Armstrong J. S. (2011). Comparing face-to-face meetings, nominal groups, Delphi and prediction markets on an estimation task. *International Journal of Forecasting*.

[36] Wei L., Zewei Z., Hao Z. (2013). Construction of a prediction system for chinese materia medica hot/cold properties. *World Chinese Medicine*.

[37] Tepper S., Dejong G., Wilkerson D., Brannon R. (1995). Criteria for selection of a payment method for inpatient medical rehabilitation. *Archives of Physical Medicine and Rehabilitation*.

[38] Liu X., Li N.-S., Lv L.-S. (2013). A comparison of the performances of an artificial neural network and a regression model for GFR estimation. *American Journal of Kidney Diseases*.

[39] Lowthian J. A., Jolley D. J., Curtis A. J. (2011). The challenges of population ageing: Accelerating demand for emergency ambulance services by older patients, 1995–2015. *Medical Journal of Australia*.

[40] Chi C.-L., Street W. N., Wolberg W. H. (2007). Application of artificial neural network-based survival analysis on two breast cancer datasets. *AMIA Annual Symposium Proceedings*.

[41] Çolak M. C., Çolak C., Kocatürk H., Sağiroğlu Ş., Barutçu I. (2008). Predicting coronary artery disease using different artificial neural network models. *The Anatolian Journal of Cardiology*.

[42] Peng S.-Y., Peng S.-K. (2008). Predicting adverse outcomes of cardiac surgery with the application of artificial neural networks. *Anaesthesia*.

[43] Bollschweiler E. H., Mönig S. P., Hensler K., Baldus S. E., Maruyama K., Hölscher A. H. (2004). Artificial neural network for prediction of lymph node metastases in gastric cancer: a phase II diagnostic study. *Annals of Surgical Oncology*.

[44] Delen D., Walker G., Kadam A. (2005). Predicting breast cancer survivability: a comparison of three data mining methods. *Artificial Intelligence in Medicine*.

[45] Jerez-Aragonés J. M., Gómez-Ruiz J. A., Ramos-Jiménez G., Muñoz-Pérez J., Alba-Conejo E. (2003). A combined neural network and decision trees model for prognosis of breast cancer relapse. *Artificial Intelligence in Medicine*.

[46] Gohari M. R., Biglarian A., Bakhshi E., Pourhoseingholi M. A. (2011). Use of an artificial neural network to determine prognostic factors in colorectal cancer patients. *Asian Pacific Journal of Cancer Prevention*.

[47] Mariani L., Coradini D., Biganzoli E. (1997). Prognostic factors for metachronous contralateral breast cancer: a comparison of the linear Cox regression model and its artificial neural network extension. *Breast Cancer Research and Treatment*.

[48] Maroco J., Silva D., Rodrigues A., Guerreiro M., Santana I., de Mendonça A. (2011). Data mining methods in the prediction of Dementia: a real-data comparison of the accuracy, sensitivity and specificity of linear discriminant analysis, logistic regression, neural networks, support vector machines, classification trees and random forests. *BMC Research Notes*.

[49] Grainger C., Griffiths R. (1994). Day surgery-how much is possible? A Delphi consensus among surgeons. *Public Health*.

[50] Kors J. A., van Bemmel J. H. (1989). The Delphi method: a review of its applications in medicine. *Medinfo*.

[51] Hickey S., Roberts H. (2004). *Ascorbate: The Science of Vitamin C*.

[52] Jones R. (2014). Forecasting conundrum: a disease time cascade. *British Journal of Health Care Management*.

[53] Surowiecki J.

[54] Starkweather D. B., Gelwicks L., Newcomer R. (1975). Delphi forecasting of health care organization. *Inquiry*.

[55] Surowiecki J. (2004). The wisdom of crowds. *Why the Many are Smarter Than the Few and How Collective Wisdom Shapes Business, Economies, Societies and Nations*.

[56] Silber I., Israeli A. A. (2012). Using information markets for pricing: the case of the airline industry. *Journal of Hospitality Marketing and Management*.

[57] Pill J. (1971). The Delphi method: substance, context, a critique and an annotated bibliography. *Socio-Economic Planning Sciences*.

[58] Thoreau H. D. (1962). *I to Myself: An Annotated Selection from the Journal of Henry D. Thoreau (Jeffrey S. Cramer, ed.)*.

[59] Nietzsche F. (1966). *Beyond Good and Evil*.

[60] Linstone H. A., Turoff M. (1975). *The Delphi Method: Techniques and Applications*.

[61] Xu Z., Xia H. O. (2008). The Delphi technique and its application in nursing research. *Journal of Nursing Science*.

[62] Bowles N. (1999). The Delphi technique. *Nursing Standard*.

[63] Riggs W. E. (1983). The Delphi technique. An experimental evaluation. *Technological Forecasting and Social Change*.

[64] Bender A. D., Strack A. E., Ebright G. W., Von Haunalter G. (1969). Delphic study examines developments in medicine. *Futures*.

[65] Mclennan J. Derivation and validation of a clinical prediction rule to predict the likelihood of massive transfusion in military major trauma.

[66] Zeng J., Rong X., Nie S. (2012). Developing an early warning indicators system for infectious diseases in Wuhan Urban area: a delphi approach. *Chinese Journal of Social Medicine*.

[67] Cook C., Brismée J.-M., Pietrobon R., Sizer P., Hegedus E., Riddle D. L. (2010). Development of a quality checklist using Delphi methods for prescriptive clinical prediction rules: the QUADCPR. *Journal of Manipulative and Physiological Therapeutics*.

[68] Sporer K. A., Craig A. M., Johnson N. J., Yeh C. C. (2010). Does emergency medical dispatch priority predict delphi process-derived levels of prehospital intervention?. *Prehospital and Disaster Medicine*.

[69] Hazard R. G., Haugh L. D., Reid S., Preble J. B., MacDonald L. (1996). Early prediction of chronic disability after occupational low back injury. *Spine*.

[70] Norcross J. C., Hedges M., Prochaska J. O. (2002). The face of 2010: a Delphi poll on the future of psychotherapy. *Professional Psychology: Research and Practice*.

[71] Hudak R. P., Brooke P. P., Finstuen K. (1994). FORECAST 2000: a prediction of skills, knowledge, and abilities required by senior medical treatment facility leaders into the 21st century. *Military Medicine*.

[72] Hosokawa M., Zweig S. (1989). Future directions in family medicine: results of a Delphi study. *Family Medicine*.

[73] Hall C. (2010). Prediction markets: issues and applications. *The Journal of Prediction Markets*.

[74] Kanter S. L. (2009). The future of academic medicine: what can academic medicine do about it?. *Academic Medicine*.

[75] Ho T.-H., Chen K.-Y. (2007). New product blockbusters: the magic and science of prediction markets. *California Management Review*.

[76] Spears B., LaComb C., Interrante J., Barnett J., Senturk-Dogonaksoy D. (2009). Examining trader behavior in idea markets: an implementation of GE's imagination markets. *The Journal of Prediction Markets*.

[77] Dahan E., Srinivasan V. (2000). Predictive power of Internet-based product concept testing using visual depiction and animation. *Journal of Product Innovation Management*.

[78] Strumpf K. (2009). Introduction to special issue on corporate applications of prediction markets. *The Journal of Prediction Markets*.

[79] Greibe J., Bugge P., Gjorup T., Lauritzen T., Bonnevie O., Wulff H. R. (1977). Long-term prognosis of duodenal ulcer: follow-up study and survey of doctors' estimates. *The British Medical Journal*.

[80] Lee K. L., Pryor D. B., Harrell F. E. (1986). Predicting outcome in coronary disease statistical models versus expert clinicians. *The American Journal of Medicine*.

[81] Chang R. W. S., Lee B., Jacobs S., Lee B. (1989). Accuracy of decisions to withdraw therapy in critically ill patients: clinical judgment versus a computer model. *Critical Care Medicine*.

[82] Polgreen P. M., Nelson F. D., Neumann G. R. (2007). Use of prediction markets to forecast infectious disease activity. *Clinical Infectious Diseases*.

[83] Rajakovich D., Vladimirov V. (2009). Prediction markets as a medical forecasting tool: demand for hospital services. *Journal of Prediction Markets*.

[84] Sato F., Shimada Y., Selaru F. M. (2005). Prediction of survival in patients with esophageal carcinoma using artificial neural networks. *Cancer*.

[85] Hui E. P., Leung L. K. S., Poon T. C. W. (2011). Prediction of outcome in cancer patients with febrile neutropenia: a prospective validation of the Multinational Association for Supportive Care in Cancer risk index in a Chinese population and comparison with the Talcott model and artificial neural network. *Supportive Care in Cancer*.

[86] Kaiserman I., Rosner M., Pe'er J. (2005). Forecasting the prognosis of choroidal melanoma with an artificial neural network. *Ophthalmology*.

[87] Orr R. K. (2001). Use of an artificial neural network to quantitate risk of malignancy for abnormal mammograms. *Surgery*.

[88] Lisboa P. J. G. (2002). A review of evidence of health benefit from artificial neural networks in medical intervention. *Neural Networks*.

[89] Sonke G. S., Heskes T., Verbeek A. L. M., De La Rosette J. J. M. C. H., Kiemeney L. A. L. M. (2000). Prediction of bladder outlet obstruction in men with lower urinary tract symptoms using artificial neural networks. *The Journal of Urology*.

[90] Simmons J. P., Nelson L. D., Galak J., Frederick S. (2009). Are crowds wise when predicting against point spreads? It depends on how you ask. *NA—Advances in Consumer Research*.

[91] Hogarth R. M. (1978). A note on aggregating opinions. *Organizational Behavior and Human Performance*.

[92] Makridakis S., Winkler R. L. (1983). Averages of forecasts: some empirical results. *Management Science*.

[93] Duru O., Bulut E., Yoshida S. (2012). A fuzzy extended DELPHI method for adjustment of statistical time series prediction: an empirical study on dry bulk freight market case. *Expert Systems with Applications*.

[94] Servan-Schreiber E., Wolfers J., Pennock D. M., Galebach B. (2004). Prediction markets: does money matter?. *Electronic Markets*.

[95] Caporale G. M., Gil-Alana L. (2013). Long memory and fractional integration in high frequency data on the US dollar/British pound spot exchange rate. *International Review of Financial Analysis C*.

[96] Arazy O., Morgan W., Patterson R. Wisdom of the crowds: decentralized knowledge construction in Wikipedia.

[97] Lorenz J., Rauhut H., Schweitzer F., Helbing D. (2011). How social influence can undermine the wisdom of crowd effect. *Proceedings of the National Academy of Sciences of the United States of America*.

[98] Page S. E. (2007). *The Difference: How the Power of Diversity Creates Better Groups, Firms, Schools, and Societies*.

[99] March J. G. (1991). Exploration and exploitation in organizational learning. *Organization Science*.

[100] Chase W. G., Simon H. A. (1973). Perception in chess. *Cognitive Psychology*.

[101] Sniezek J. A., Buckley T. (1995). Cueing and cognitive conflict in judge-advisor decision making. *Organizational Behavior and Human Decision Processes*.

[102] Mannix E., Neale M. A. (2005). What differences make a difference? The promise and reality of diverse teams in organizations. *Psychological Science in the Public Interest*.

[103] Weinstein N. D., Klein W. M. (1996). Unrealistic optimism: present and future. *Journal of Social and Clinical Psychology*.

[104] Rothman A. J., Klein W. M., Weinstein N. D. (1996). Absolute and relative biases in estimations of personal risk. *Journal of Applied Social Psychology*.

[105] Asch S. (1952). *Social Psychology*.

[106] Festinger L. (1954). A theory of social comparison processes. *Human Relations*.

[107] Galton F. (1907). Vox populi. *Nature*.

[108] Treynor J. L. (1987). Market efficiency and the bean jar experiment. *Financial Analysts Journal*.

[109] Hastie R., Kameda T. (2005). The robust beauty of majority rules in group decisions. *Psychological Review*.

[110] Sunstein C. R. (2006). *Infotopia: How Many Minds Produce Knowledge*.

[111] Kugler T., Kausel E. E., Kocher M. G. (2012). Are groups more rational than individuals? A review of interactive decision making in groups. *Wiley Interdisciplinary Reviews: Cognitive Science*.

[112] Radner R. (1996). Bounded rationality, indeterminacy, and the theory of the firm. *The Economic Journal*.

[113] Paton D., Siegel D. S., Williams L. V. (2009). The growth of gambling and prediction markets: economic and financial implications. *Economica*.

